# Capnocytophaga sputigena Tonsillitis in a Patient With Acute Myeloid Leukemia

**DOI:** 10.7759/cureus.56551

**Published:** 2024-03-20

**Authors:** Ethan Heh, Jesse C Allen, Mark Raynor, Rivers A Hock, Diego P Peralta

**Affiliations:** 1 Infectious Diseases, Paul L. Foster School of Medicine, Texas Tech University Health Sciences Center El Paso, El Paso, USA; 2 Infectious Diseases, El Paso VA Health Care System, El Paso, USA

**Keywords:** peritonsillar abscess, aml, leukemia, acute myeloid leukemia, tonsillitis, sputigena, capnocytophaga

## Abstract

*Capnocytophaga sputigena* is a gram-negative facultatively anaerobic, capnophilic bacterium typically residing in the human oropharyngeal flora. This opportunistic pathogen can cause a wide range of infections, from bacteremia to septic abortion. However, it is exceedingly rare for a patient to present with tonsillitis due to *C.* *sputigena*. Herein, we discuss the presentation, hospital course, and clinical trajectory of a patient experiencing complications of tonsillitis related to* C. sputigena *in the context of acute myeloid leukemia. Additionally, we delve into the treatment approaches and challenges in managing this particular pathogen.

## Introduction

*Capnocytophaga *is a genus of gram-negative facultatively anaerobic, capnophilic bacteria that typically inhabit the oropharyngeal flora. There are many species of *Capnocytophaga*, including *C.* *ochracea*, *C.* *gingivalis*, *C.* *sputigena*, *C.* *leadbetteri*, *C.* *haemolytica*, *C.* *granulosa*, *C.* *cynodegmi*, *C.* *canis*, and *C.* *canimorsus*. They are opportunistic pathogens associated with periodontal disease and animal bites that can infect immunocompetent and immunocompromised hosts [1]. This genus can cause many infections, including abscesses, bacteremia, pneumonia, empyema, septic abortion, mucositis, and more [2-5]. Specifically, *C.* *canimorsus*, an organism residing in the saliva of dogs and cats, can be transmitted via penetrating bites and can cause septic shock in immunocompetent patients [6]. In comparison, *C.* *sputigena *is present in the normal human oral flora, causing infections in immunocompromised hosts. Infections by *C.* *sputigena *are relatively rare and have been seen in those with hematological malignancies. Further, the human-oral-associated *Capnocytophaga *species (*C.* *sputigena*, *C.* *ochracea*, and *C.* *gingivalis*) have worse outcomes with an overall mortality at six months of 36.4% compared to 6.2% in *C.* *canimorsus *[1]. So far, there have been only 11 reported cases of *C.* *sputigena *causing bacteremia in a patient with hematological malignancy without any presentation of tonsillitis [7-11]. Herein, we report the first case of acute myeloid leukemia complicated by tonsillitis due to *C.* *sputigena*.

## Case presentation

This is the case of a 78-year-old man with acute myeloid leukemia receiving venetoclax, chemotherapy-induced pancytopenia, and thrombocytopenia dependent on platelet transfusions who presented to the emergency department with a three-day history of sore throat, dysphagia to solids, right-sided facial pain, and neck pain. He was referred after being evaluated in the outpatient clinic due to worsening symptoms and concerns of possible peritonsillar abscess. 

On presentation, the patient had a temperature of 37.9℃ but was hemodynamically stable. A complete physical exam revealed a thin male in no acute distress, exhibiting bilateral tonsillar enlargement with exudates and without uvular shift and poor dentition. The patient displayed non-labored respirations and normal speech. Initial blood work revealed severe pancytopenia and normocytic anemia (Table 1).

**Table 1:** Initial lab work results. *: abnormal lab value

Tests	Normal range	Results
White blood cells	4.5-11.0 x 10^3^/µL	0.86 x 10^3^/µL*
Neutrophils	2.0-7.8 x 10^3^/µL	0.00 x 10^3^/µL
Hemoglobin	12.0-15.0 g/dL	6.6 g/dL*
Hematocrit	40-54%	20.6%*
Mean corpuscular volume	82-98 fL	90.0 fL
Platelets	150-450 x 10^3^/µL	17 x 10^3^/µL*
Sodium, serum	135-145 mmol/L	136 mmol/L
Potassium, serum	3.5-5.1 mmol/L	4.7 mmol/L
Chloride, serum	98-107 mmol/L	102 mmol/L
Glucose	74-106 mg/dL	106 mg/dL
Creatinine	0.52-1.04 mg/dL	0.7 mg/dL
Blood urea nitrogen	7-17 mg/dL	16 mg/dL

**Figure 1:**
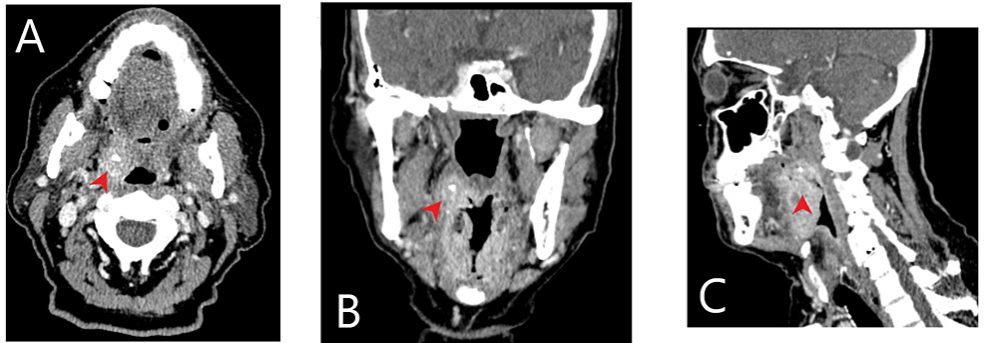
CT of the soft tissue neck with contrast showing peritonsillar fluid collections on the right side. Red arrowhead: peritonsillar fluid collection. A) Axial. B) Coronal. C) Sagittal

Diagnostic measures, including a soft tissue neck CT, chest X-ray, and blood cultures, were obtained. The neck CT revealed asymmetric right tonsillar enlargement/tonsillitis and peritonsillar fluid collections. The right collection was bigger than the left (Figure 1). The chest X-ray demonstrated no thoracic abnormalities. The patient was empirically started on intravenous vancomycin and cefepime and was subsequently admitted with the working diagnosis of neutropenic fever and severe tonsillitis.

During the hospitalization, consultation with oncology prompted the addition of acyclovir and fluconazole for herpes simplex virus and *Candida* prophylaxis, respectively. Due to concerns about pancytopenia, the patient's chemotherapy was temporarily halted and scheduled for resumption in three days. ENT consultation concluded surgical intervention was unnecessary, given the improved clinical status and non-drainable abscess. 

Despite clinical improvement, the patient remained fatigued and intermittently febrile. Infectious disease consultation resulted in adjustment to the antimicrobial regimen by maintaining vancomycin while transitioning from cefepime to piperacillin-tazobactam to add coverage of oropharyngeal anaerobes. Fluconazole was substituted with posaconazole, given persistent neutropenia, and acyclovir was continued. Chemotherapy was restarted with azacitidine and venetoclax.

On the sixth day, blood cultures returned positive with the growth of *Capnocytophaga sputigena*, leading to the discontinuation of vancomycin. However, one day later, the patient developed profuse watery diarrhea and was found to be *Clostridioides difficile *antigen-positive and toxin-negative. Treatment was initiated with oral vancomycin. Two days later, a nonpruritic maculopapular rash appeared on the abdomen and chest, attributed to antibiotic use, specifically piperacillin-tazobactam. This prompted the discontinuation of piperacillin-tazobactam and the initiation of meropenem for six days. His antibiotic course and rationale are displayed below (Table 2).

**Table 2:** Antimicrobial course. PO: oral; IV: intravenous

Antimicrobial regimen	Hospital day	Rationale
IV vancomycin cefepime	1	Empiric treatment
Addition of acyclovir and fluconazole	2	Viral and fungal prophylaxis
Changed cefepime to piperacillin-tazobactam	5	Coverage for oropharyngeal anaerobes
Switched fluconazole to posaconazole	5	Mold prophylaxis given prolonged neutropenia
Decreased posaconazole dose	6	Possible interactions with the chemotherapy regimen
Discontinued IV vancomycin	7	Blood cultures revealed *Capnocytophaga sputigena*
Started on PO vancomycin	8	*Clostridioides difficile* diarrhea
Switched from piperacillin-tazobactam to meropenem	9	A new-onset maculopapular rash was suspected to be piperacillin-tazobactam-induced

The remaining clinical course was relatively uncomplicated, with the patient maintaining an afebrile state until discharge. Management of acute myeloid leukemia involved transfusion support and resumption of the patient's chemotherapy regimen. The maculopapular rash and *C.* *difficile*-associated diarrhea resolved but required continued oral vancomycin for 16 days. He was continued on mold and viral prophylaxis until discharge for a total of 25 days until neutropenia resolved.

## Discussion

An extensive literature search in the PubMed and Google Scholar databases identified only 11 cases of *C. sputigena *bacteremia in a patient with hematologic malignancy. Of those cases, none has ever been complicated by tonsillitis. The details of those cases are included in Table 3.

**Table 3:** Previous cases of Capnocytophaga sputigena bacteremia in patients with a hematologic malignancy. NOS: not otherwise specified

Author(s) and year	Study type	Age and sex	Hematological malignancy	Complications	Antibiotic treatment	Outcome
Garcia Lozano et al., 2012 [7]	Case report	44-year-old man	Non-Hodgkin lymphoma type T specified	Bacteremia	Imipenem and vancomycin	Death
Isabel 2019 [8]	Case report	17-year-old man	Lymphoma/lymphoblastic leukemia	Mucositis and bacteremia	Meropenem	Resolved bacteremia
Kim et al., 2014 [9]	Case report	40-year-old man	Chronic lymphocytic leukemia	Bacteremia	Piperacillin-tazobactam	Resolved bacteremia
Mendes et al., 2020 [10]	Case report	23-year-old man	Hodgkin lymphoma	Mucositis and bacteremia	Meropenem linezolid and piperacillin-tazobactam	Resolved bacteremia
		50-year-old man	Peripheral T-cell NOS lymphoma	Mucositis and bacteremia	Meropenem linezolid and piperacillin-tazobactam	Resolved bacteremia
Bonatti et al., 2003 [11]	Case series	37-year-old man	Chronic myelogenous leukemia	Mucositis and bacteremia	Imipenem-cilastatin, penicillin G	Death
		34-year-old woman	Chronic myelogenous leukemia	Mucositis and bacteremia	Piperacillin-tazobactam	Death
		32-year-old woman	Acute myelogenous leukemia	Mucositis and bacteremia	Imipenem-cilastatin	Resolved bacteremia
		44-year-old woman	Acute myelogenous leukemia	Mucositis and bacteremia	Cefamandole, tobramycin	Death
		22-year-old woman	Recurrent Hodgkin disease	Mucositis and oral thrush and bacteremia	Imipenem-cilastatin	Resolved bacteremia
		46-year-old man	Chronic myelogenous leukemia	Mucositis and bacteremia	Imipenem-cilastatin	Resolved bacteremia

Our case exemplifies the unique presentation that *C.* *sputigena *can take in a patient with a hematological malignancy receiving chemotherapy. In many cases, oral mucositis and ulceration occur during chemotherapy treatment, creating an increased risk of the oral cavity to bloodstream infection. For example, the studies by Kim et al. [9] and Mendes et al. [10] showed bacteremia after hematopoietic stem cell transplantation (HSCT) or bone marrow transplantation. With our patient on venetoclax, neutropenia almost always occurs (84%), and microbiologically documented infections (30.5%) are commonly seen [12]. However, the literature has never reported *C.* *sputigena *infection of the tonsils.

Further, the antibiotic treatment of *C.* *sputigena *varies widely, as evidenced by Table 3. This is due to the varying β-lactamase resistance and susceptibilities of this species. A study by Ehrmann et al. [13] found that 22% of *C.* *sputigena *have resistance to β-lactam and macrolide-lincosamide-streptogramin antibiotics. This species typically harbors the blaCfxA or blaCSP-1 genes, increasing the resistance to amoxicillin, amoxicillin-clavulanic acid, and third-generation cephalosporins. Moreover, in vitro activities against *Capnocytophaga *spp. revealed that linezolid, imipenem, and some β-lactamase inhibitor combinations exhibit antimicrobial susceptibility [14]. In a study by Jolivet-Gougeon et al. [15], in vitro piperacillin-tazobactam and carbapenems showed lower minimum inhibitory concentrations (MICs) against *Capnocytophaga *spp. (2 μg/ml and 0.5 μg/ml needed for 90% inhibition, respectively), while cephalosporins had much higher MICs (8-64 μg/ml needed for 90% inhibition). This is important in guiding empiric antibiotic management once blood cultures reveal *Capnocytophaga *spp. Further, if complications arise regarding the antibiotic side effects, such as a maculopapular rash secondary to piperacillin-tazobactam, the clinician can adjust to a carbapenem appropriately.

## Conclusions

Our case study shows that in a patient with neutropenic fever and hematological malignancy, the presentation of *C.* *sputigena *can vary greatly. Tonsillitis secondary to this pathogen has never been reported in the literature, and its presentation and management can be challenging. With suspected oropharyngeal and maxillofacial infections, it is essential to remember anaerobic coverage and the efficacious antibiotics in treating *C.* *sputigena*.
